# Terson’s syndrome: an important differential diagnosis of subarachnoid hemorrhage

**DOI:** 10.1590/0100-3984.2016.0053

**Published:** 2017

**Authors:** Ana Paula Alves Fonseca, Marcos Rosa Júnior

**Affiliations:** 1 Hospital Universitário Cassiano Antônio Morais da Universidade Federal do Espírito Santo (HUCAM-UFES), Vitória, ES, Brazil.

**Figure 1:**
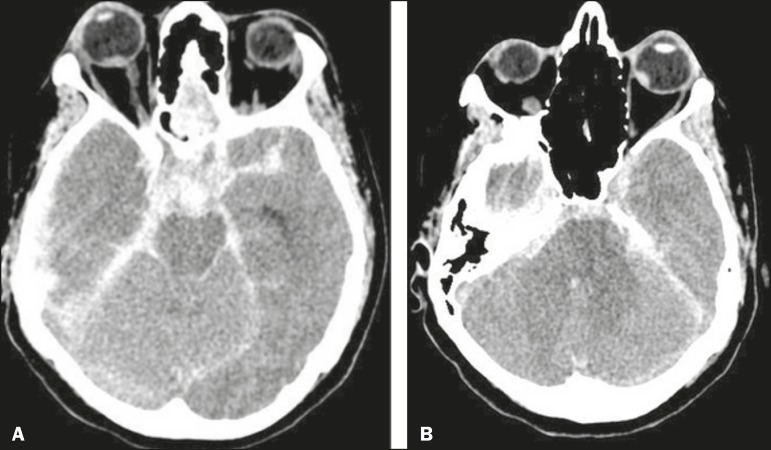
A: TC demonstrando sinais de hemorragia subaracnoide e hemorragia intraocular direita, como foco espontaneamente hiperatenuante na porção posterior do globo ocular direito. B: TC mostrando hemorragia intraocular à esquerda.

Dear Editor,

A 42-year-old female patient presented to the emergency room with severe headache and hypertensive urgency (blood pressure, 220/110 mmHg), progressing to left hemiparesis, right anisocoria, and a decreased level of consciousness, with a Glasgow Coma Scale score of 4. Computed tomography (CT) of the brain showed acute subarachnoid hemorrhage (Fisher grade 4), due to rupture of an aneurysm in the anterior circulation, together with signs of bilateral intraocular hemorrhage (Figure 1). Those findings are consistent with a diagnosis of Terson’s syndrome.

Terson’s syndrome was initially described as vitreous hemorrhage secondary to acute subarachnoid hemorrhage, although recent studies have shown that it can also result from traumatic brain injury or even nontraumatic intracerebral hemorrhage^(1)^. Originally described in 1900 by Albert Terson, the syndrome has an incidence of 2.6–27.0% in the context of subarachnoid hemorrhage due to a ruptured aneurysm^(2-4)^. Although the etiology of the syndrome is controversial, it has been attributed to a rapid increase in venous or intracranial pressure, which causes rupture of the peripapillary capillaries of the retina or results in compression of the central retinal vein, thus decreasing retinal venous drainage, promoting stasis, and provoking hemorrhage^(5)^.

The diagnosis of intraocular hemorrhage is more accurately confirmed by ophthalmoscopy, although CT can suggest it, with an estimated sensitivity of 66%. The changes seen most frequently are retinal thickening and hyperattenuating nodules overlying the optic disc^(6)^.

Terson’s syndrome most often occurs in patients with severe neurological disease, a Glasgow Coma Scale £ 8, and aneurysmal subarachnoid hemorrhage with a Fisher score ³ 3 at presentation. It is also of note that the rates of morbidity and mortality are high among such patients. In the sample studied by Fountas et al.^(7)^, the mortality was 28.6% among the patients with intraocular hemorrhage, compared with only 2.0% among those without.

Terson’s syndrome is not an uncommon condition, perhaps being underdiagnosed. Given the prognostic implications of this diagnosis for morbidity and mortality, as well as the potential for secondary ocular lesion, it is of extreme relevance to radiologists and other medical professionals, especially in the context of acute subarachnoid hemorrhage^(8)^ but also in other forms of intracranial hemorrhage.
